# Generational Views of Life Challenges and the Aging Process: Influence of Relationship Type, Frequency, Experience

**DOI:** 10.1093/geroni/igab046.1551

**Published:** 2021-12-17

**Authors:** Mary Pagan

**Affiliations:** SUNY Oswego, Baldwinsville, New York, United States

## Abstract

This study, conducted within a higher education setting, examines and compares views of younger adults (age 20-24) and older adults (age 65-97) in two major areas; life challenges and aging processes. Are they complimentary, neutral, or opposing? We investigate views and the potential association to relationship type (family relation, close contact, casual), frequency (times interacting per month) including mode of communication (face-to-face, phone, visual technology), and narrative of overall experience. A mixed-method approach incorporated convenience survey data and extensive in-depth interviews. Data collection instruments were designed and conducted by students in an upper-division wellness and aging course at SUNY Oswego. Students partnered with older adult study participants. Participant (n=80) inquiries centered on life challenges (COVID, adversity, loss, discrimination/bias, regrets) and aging processes (views on aging, life expectancy, changes in health). The study also examines the impact of the 3-credit course on aging views; specifically did they change their life expectancy choice (years) from the week one by the end of the course.

